# Peripheral nerve magnetic resonance imaging

**DOI:** 10.12688/f1000research.19695.1

**Published:** 2019-10-28

**Authors:** Yongsheng Chen, E. Mark Haacke, Jun Li

**Affiliations:** 1 Department of Neurology, Wayne State University School of Medicine, Detroit, MI, 48201, USA; 2 Department of Radiology, Wayne State University School of Medicine, Detroit, MI, 48201, USA; 3 Center for Molecular Medicine & Genetics, Wayne State University School of Medicine, Detroit, MI, 48201, USA; 4 Department of Biochemistry, Microbiology and Immunology, Wayne State University School of Medicine, Detroit, MI, 48201, USA; 5 John D. Dingell VA Medical Center, Detroit, MI, 48201, USA

**Keywords:** Peripheral Nervous System, Peripheral Nerves, Sciatic Nerve, Magnetic Resonance Imaging, Peripheral Neuropathy, Charcot-Marie-Tooth Disease

## Abstract

Magnetic resonance imaging (MRI) has been used extensively in revealing pathological changes in the central nervous system. However, to date, MRI is very much underutilized in evaluating the peripheral nervous system (PNS). This underutilization is generally due to two perceived weaknesses in MRI: first, the need for very high resolution to image the small structures within the peripheral nerves to visualize morphological changes; second, the lack of normative data in MRI of the PNS and this makes reliable interpretation of the data difficult. This article reviews current state-of-the-art capabilities in *in vivo* MRI of human peripheral nerves. It aims to identify areas where progress has been made and those that still require further improvement. In particular, with many new therapies on the horizon, this review addresses how MRI can be used to provide non-invasive and objective biomarkers in the evaluation of peripheral neuropathies. Although a number of techniques are available in diagnosing and tracking pathologies in the PNS, those techniques typically target the distal peripheral nerves, and distal nerves may be completely degenerated during the patient’s first clinic visit. These techniques may also not be able to access the proximal nerves deeply embedded in the tissue. Peripheral nerve MRI would be an alternative to circumvent these problems. In order to address the pressing clinical needs, this review closes with a clinical protocol at 3T that will allow high-resolution, high-contrast, quantitative MRI of the proximal peripheral nerves.

## Introduction

### The peripheral nervous system

In order to execute commands from the central nervous system (CNS) (consisting of the brain and spinal cord), humans need the peripheral nervous system (PNS) to provide a communication route from their “external devices” such as sensory organs or muscles to the brain. Thus, these peripheral nerves are designed to travel between the brain, through the spinal cord, and eventually to the organs outside of the cranial space or spinal canal ^1^.

Peripheral nerves are well-organized tubular structures running from the spinal cord/brain to the cranial tissues and the extremities. For simplification, this review will omit the cranial nerves. The spinal cord extends the nerve fibers from its ventral column, called ventral roots (primarily motor nerve fibers), and from its dorsal column, called dorsal roots (primarily sensory nerve fibers). Ventral and dorsal roots meet together and are encased by epineurial tissues right before they exit the spinal canal at the neural foramen. These nerves from different levels of the neural foramen are intermingled to form either the brachial plexus in the neck or lumbosacral plexus in the lower back and branched into peripheral nerves in the extremities, such as the ulnar, median, radial, femoral, and sciatic nerves ^1^.

**Figure 1.**
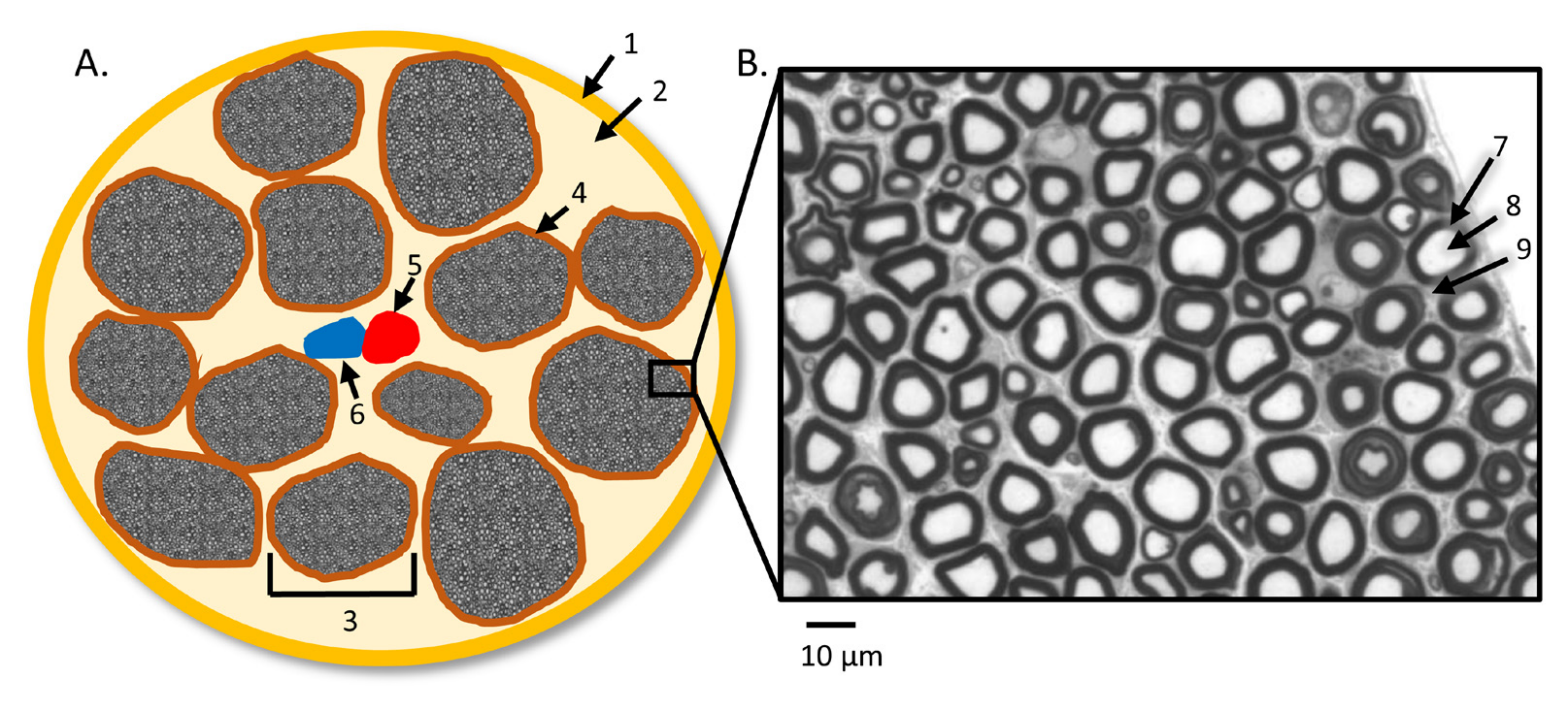
Peripheral nerve cross-sectional anatomy. ( **A**) Illustration of peripheral nerves’ cross-sectional anatomy with the presence of intraneural blood vessels: (1) epineurium, (2) lipid-equivalent connection tissues, (3) individual nerve fascicle, (4) perineurium, (5) artery, and (6) vein. ( **B**) Representative myelinated nerve fibers under light microscopy from a control sciatic nerve ^8^, stained with Toluidine blue: (7) myelin sheath, (8) axon, and (9) endoneurium. The picture in B was tailored from Figure 1A of Li *et al*. ^8^.

The structure of the peripheral nerves can be well recognized on a transverse section, as shown in Figure 1. Here, the outermost layer is the fibrous connective tissue referred to as the epineurium, and inside the epineurium, individual nerve fibers are organized into individual bundles, called fascicles, by another layer of fibrous connective tissue called perineurium. The number and size of human peripheral nerve fascicles vary considerably; the size typically ranges from 0.1 to 1 mm in diameter ^2^. There are two types of nerve fibers: myelinated and unmyelinated. The former is made up of layers of Schwann cell membranes (myelin) in segments (called internodes) to wrap axons concentrically ^3^. The segments are separated by punctuate gaps, called nodes of Ranvier, where the axon is denuded of myelin. Myelinated nerve fibers are separated from each other by another layer of connective tissue, called endoneurium, serving as part of the blood–nerve barrier which prevents molecules from crossing the blood into the endoneurial fluid. The non-myelinated nerve fibers, called C fibers, are numerous and run alongside the myelinated axons. These axons are circled by one layer of Schwann cell membrane without forming myelin. These unmyelinated axons are grouped into what is known as a Remak bundle. There are also abundant blood vessels within the nerve epineurium and perineurium. Between the nerve axon and the endoneurium, there is a low-protein liquid, called endoneurial fluid. The myelinated nerve axon propagates electrical signals, the action potential, between the CNS and distant organs. The myelin insulates axons and permits salutatory conduction of action potentials with greater velocity than that in non-myelinated axons ^1– 5^.

### Peripheral neuropathy

Diseases damaging peripheral nerves are collectively called peripheral neuropathy, affecting either multiple nerves (called polyneuropathy) or only one nerve (called mononeuropathy). Polyneuropathy is a group of diseases affecting peripheral nerves in roughly symmetric areas in bilateral limbs. It is clinically characterized by muscle weakness, numbness, and burning pain in the hands and feet. The disease may progress to the proximal arms and legs (length-dependent process) and sometimes to other parts of the body, such as autonomic nerves. Collectively, these diseases are highly prevalent and affect about 8% of people above 55 years of age ^6^. Common types of polyneuropathy include diabetic peripheral neuropathy (DPN), Guillain–Barré syndrome, chronic inflammatory demyelinating polyneuropathy (CIDP), human immunodeficiency virus (HIV)-associated neuropathy, alcoholic neuropathy, and paraproteinemic neuropathy.

When the disease is caused by monogenic mutations, it is called Charcot–Marie–Tooth disease (CMT). Like other types of polyneuropathy, CMT is affected mainly by two key pathological lesions: demyelination (or dysmyelination if abnormally formed myelin occurs during development) and axonal degeneration. A major group of CMT diseases are caused by abnormalities in the myelin sheath that are classified as type 1 (CMT1) with autosomal dominant inheritance. The most common subtype of CMT1 is CMT1A. It is caused by duplication of chromosome 17p12, a DNA segment containing the peripheral myelin protein-22 ( *PMP-22*) gene ^7^. CMT1B is caused by genetic mutations of the myelin protein zero ( *P0*) gene ^8^. The other major type of CMT, CMT2, is affected predominantly by axonal degeneration. CMT2A is the most common subtype of CMT2 and is caused by missense mutations in the *Mitofusin-2* gene, which encodes a protein regulating mitochondrial fusion ^9^. All CMT patients with x-linked inheritance are classified as CMTX. All CMT patients with autosomal recessive inheritance are grouped as CMT4.

Peripheral nerve injury has a high prevalence, affecting about 3% in the trauma population, whose trauma often is caused by motor vehicle accidents ^10^. There are three major types of peripheral nerve injuries described by Seddon ^11^ and Sunderland ^12^: neurapraxia, axonotmesis, and neurotmesis. Neurapraxia is primary demyelination with a reversible conduction block in myelinated nerve fibers. Axonotmesis results in axonal degeneration. Because the neuronal cell body is not damaged, the axon can regenerate at a very slow rate (about 1 mm per day). The distal end of the axon separated from the cell body undergoes Wallerian degeneration. The neurotmesis is a mixture of demyelination and axon loss with disruption of endoneurium, perineurium, or epineurium. When endoneurium is damaged, the axon may regrow; however, poor growth is expected in those nerves with perineurial damage. When the epineurium is damaged, usually there will be no regrowth ^5, 13^.

### Current diagnostic tools

Irrespective of causes or types of polyneuropathies, nerves are afflicted mainly by two kinds of lesions: axonal degeneration and de-/dysmyelination. Ascertaining the pathology has been crucial in diagnosing and treating polyneuropathies. For instance, demyelinating polyneuropathies are often responsive to immunomodulatory therapies, but axonal polyneuropathies have no effective treatment to date. Moreover, quantification of axonal/myelin pathologies is required to accurately track the progression of polyneuropathies, which is often critical for clinical trials. Traditionally, these pathologies have to be evaluated by sural nerve biopsy, which is an invasive procedure involving surgery to remove the nerve in the leg. Nerve biopsy is not suitable for longitudinal studies since repetitive surgeries are practically prohibitive. Peripheral nerves can also be assessed by nerve conduction studies (NCSs). Demyelinating polyneuropathy is indicated if NCSs show a slowed conduction velocity, temporal dispersion, and conduction block ^14^. In contrast, axonal polyneuropathy has normal or minimally slowed conduction velocity but reduced amplitude of compound nerve action potentials ^15^. Although NCS has been a very helpful tool, nerves in many patients may become non-responsive in distal limbs because of severe degeneration. This “floor” effect can prevent NCSs from providing meaningful information.

Moreover, nerves residing in the deep tissues of proximal limbs are usually not accessible to NCSs; thus, pathologies in the proximal nerves may be missed. There are many peripheral nerve diseases primarily affecting proximal nerves, such as plexopathies, nerve injuries, or demyelination to the proximal locations. High-resolution ultrasound is an alternate approach to image the peripheral nerves. Ultrasound provides real-time imaging of the peripheral nerve for a long-axis view and the ability to do a contralateral comparison. However, the imaging quality relies largely on the technician’s experience, which affects intersubject reliability. As in NCS, proximal nerves that are deep in the tissue are usually not visible or accessible to ultrasound ^16, 17^.

More recently, magnetic resonance imaging (MRI) has been shown to provide rich image contrast, high resolution, and more quantitative features that could be viable biomarkers for peripheral nerve pathology in patients with neuropathies ( Table 1). Although MRI has been extensively utilized to reveal pathological changes in the CNS, it has been underutilized in human PNS studies *in vivo*. This is often due to two key reasons: first, the small structures in the nerves require high-resolution imaging to visualize morphological changes; second, there is little normative data for MRI in the PNS and this makes a reliable interpretation of the data difficult. On the other hand, many MRI tissue properties, such as proton density (PD), longitudinal (T1) and transverse (T2) relaxations, susceptibilities, and magnetization transfer (MT), have been well established for human brain imaging *in vivo*. These quantitative measurements have improved MRI image contrast between healthy and pathologic tissues. In particular, these MRI properties offer better visualization of the processes during inflammation, infarction, demyelination, and axonal degeneration ^18^ but these quantitative imaging modalities have not yet been well tested in the peripheral nerves.

**Table 1.** Magnetic resonance imaging (MRI) findings in peripheral neuropathies in the literature.

Reference	MRI methods	B0	MRI targets	Cohorts	Findings
Dortch *et al*. ^19^ *Neurology* (2014)	MTR	3T	SN at mid thigh	CMT1A (n = 10) CMT2A (n = 3) HNPP (n = 3) HC (n = 21)	MTR significantly decreased in CMT1A and CMT2A relative to HC. MTR decreases significantly related to disability scores. No significant difference between CMT1A and CMT2A was found. Proximal nerve volumetric MTR was repeatable from the inter-scan and inter-rater reliability analyses.
Shibuya *et al*. ^20^ *Annals of neurology* (2015)	MRN, STIR	1.5T	Nerve roots and trunks at arms	CIDP (n = 33) DC (n = 32)	Nerve hypertrophy was found in 88% of patients with CIDP and 71% of patients with CMT but not in any DC patients. According to the clinical subtype of CIDP, typical patients with CIDP showed symmetric and root-dominant hypertrophy.
Morrow *et al*. ^21^ *The Lancet Neurology* (2016)	FF, T2, MTR	3T	Muscle at thigh and calf	CMT1A (n = 18) IBM (n = 18) HC (n = 37)	Whole muscle FF increased significantly during the 12-month follow-up at the calf level but not at the thigh level in CMT1A and at the calf level and thigh level in IBM. FF correlated with clinical scores in both CMT1A and IBM cohorts. T2 increased and MTR decreased consistently with FF increases but more variably.
Chhabra *et al*. ^22^ *Journal of Magnetic Resonance Imaging* (2016)	MRN, DTI	3T	Whole body	CMT (n = 11) HC (n = 9)	Nerve hyperintensity and thickenings were significantly different for both brachial and lumbosacral plexus in CMT in comparison with HC. SN and FN size increases and FA decreases were significant in CMT in comparison with HC. Transverse dimensions of C8, L5, and S1 nerve roots and SN were the most accurate diagnostic performance to tell disease.
Kronlage *et al*. ^23^ *Journal of neurology* (2017)	MRN, T2	3T	LS plexus, arm, and leg	CIDP (n = 18) HC (n = 18)	Increased nerve CSA and T2W signal intensity were significant in CIDP in comparison with HC. ROC revealed the best diagnostic accuracy for CSA of the LS plexus (AUC = 0.88) and T2W signal intensity of the SN (AUC = 0.88). CSA correlated with NCS parameters of SN and MN. T2W signal intensity correlated with F wave latency of SN but not MN. T2 quantifications indicated that T2W hyperintensity in CIDP was from increased PD but not increased T2.
Vaeggemose *et al*. ^24^ *Muscle & Nerve* (2017)	MRN, DTI, T2, PD	3T	SN at mid thigh and TN at mid calf	CMT1A (n = 15) HC (n = 30)	In the CMT1A cohort, compared with HC, T2 had no difference at both SN and TN. FA decreases and ADC increases were significant at both SN and TN. PD increases were significant at SN but not at TN. FA was significantly correlated to NCV in SN.
Lichtenstein *et al*. ^25^ *Annals of clinical and translational neurology* (2018)	T2W, DTI, FF	3T	Muscle and SN at mid thigh	CIDP (n = 11) HC (n = 11)	In the CIDP cohort, compared with HC, CSA increases on T2W and FA decreases on DTI both for SN and increased FF in muscles were significant at baseline and 6-month follow-up. No significant longitudinal changes were observed in the 6-month follow-up for SN’s CSA and FA and muscles’ FF. SN’s CSA was positively correlated with muscle FF at the 6-month point. No significant correlations were found between either SN’s FA or muscles’ FF with clinical and NCS measurements.
Jende *et al*. ^26^ *Annals of neurology* (2018)	MRN, T2W	3T	SN at mid thigh	T1D (n = 36) T2D (n = 84)	T2W hyperintense lesions correlated negatively with TN’s CMAP and PN’s NCV and positively with NDS, NSS, and HbA1c level. T2W hypointense lesions correlated positively with NDS, NSS, and serum triglycerides and negatively with serum HDL. For DPN in T1D, elevated values of T2W hyperintense lesions and HbA1c were found in comparison with T2D. For DPN in T2D, elevated T2W hypointense lesions and triglycerides and lower serum HDL were found in comparison with T1D.
Cornett *et al*. ^27^ *Muscle & Nerve* (2018)	T1W, FF	1.5T	Calf	CMT (n = 55)	In all childhood CMT patients (n = 55), scaled muscle and fat volumes significantly correlated with measures of strength and gait variables. Muscle volume was significantly correlated with disability. Lower muscle volume and intramuscular fat accumulation were both significantly associated with reduced dorsiflexion strength, impaired gait profile score, and reduced internal hip rotation at 25% of the gait cycle.
Schmid *et al*. ^28^ *Investigative radiology* (2018)	DTI SSFP	7T	Wrist	CTS (n = 8) HC (n = 6)	Nerve fascicles of MN and UN at the wrist level can be visualized clearly on SSFP high-resolution image (0.2 × 0.2 mm ^2^) at 7T. CSA of MN was significantly larger at the hamate bone in CTS than HC. MN’s FA values were significantly decreased in CTS compared with HC, but there was no difference for UN which is not directly affected in CTS. RD was significantly higher at the pisiform and hamate bone, but not for MD and AD, in CTS compared with HC.

AD, axial diffusivity; ADC, apparent diffusion coefficient; AUC, area under receiver operating characteristic curve; CIDP, chronic inflammatory demyelinating polyneuropathy; CMAP, compound motor action potential; CMT, Charcot–Marie–Tooth disease; CSA, cross-sectional area; CTS, carpal tunnel syndrome; DC, disease control; DPN, diabetic peripheral neuropathy; DTI, diffusion tensor imaging; FA, fractional anisotropy; FF, fat fraction; FN, femoral nerve; HC, healthy control; HDL, high-density lipoprotein; HNPP, hereditary neuropathy with liability to pressure palsies; IBM, inclusion body myositis; LS, lumbosacral; MD, mean diffusivity; MN, median nerve; MRN, magnetic resonance neurography; MTR, magnetization transfer ratio; NCS, nerve conduction study; NCV, nerve conduction velocity; NDS, neuropathy disability score; NSS, neuropathy symptom score; PD, proton density; PN, peroneal nerve; RD, radial diffusivity; ROC, receiver operating characteristic analysis; SN, sciatic nerve; SSFP, steady-state free precession; STIR, short-tau inversion recovery; T1D, type 1 diabetes; T2D, type 2 diabetes; T2, transverse relaxation time; TN, tibial nerve; UN, ulnar nerve.

This article reviews the current peripheral nerve MRI techniques and studies mainly in assessing various peripheral neuropathies and closes with a clinical protocol at 3T that will allow high-resolution, high-contrast, quantitative MRI of the proximal peripheral nerves.

## Peripheral nerve magnetic resonance imaging

### Challenges in imaging the peripheral nerves

MRI offers a wide variety of contrasts coming from the tissue properties, water motion either microscopically via diffusion or macroscopically via blood flow and magnetic susceptibility, to name just a few that are of interest to imaging the PNS.

Tissue properties include water content or PD, T1, and T2 spin relaxation. Using these tissue characteristics, one can obtain proton density–weighted (PDW), T1-weighted (T1W), and T2-weighted (T2W) images by varying the timing parameters such as echo time (TE) and repetition time (TR), with either a gradient echo (GE)-based sequence or a spin echo (SE)-based sequence. By adding another radiofrequency (RF) pulse prior to the excitation, one can null the signal in certain tissues at the imaging readout period. These techniques are known as fluid-attenuated inversion recovery (FLAIR) for nulling bulk water and short-tau inversion recovery (STIR) for nulling fatty tissues. Note that with a GE acquisition, the image contrast varies from PDW to T1W by changing the flip angle through which the spins are tipped, from small to large angles. These methods are qualitative in nature, but tissue properties can be quantified by varying the imaging parameters and then reconstructing quantitative maps from this set of images. However, to accomplish truly quantitative maps of T1, T2, T2*, and PD, the calibrations of main field (B _0_) inhomogeneity as well as RF transmit (B _1_^+^) and receiver (B _1_^−^) fields must be included.

Water motion exists everywhere in the human body in either a macroscopic or microcosmic form. Typical applications of the former type of water motion are imaging the vasculature of the human body, such as MR angiography and venography. These hemodynamic characteristics can also be quantified by using either the magnitude of the signal or the phase information. These macro-water motions are largely to be avoided in the purpose of reducing image artifacts caused by motion. On the other hand, for molecular-level water motion, methods such as diffusion-weighted imaging (DWI), diffusion tensor imaging (DTI), and MT all play significant roles in today’s neurological and musculoskeletal imaging of the CNS and PNS.

The other major contrast mechanism, magnetic susceptibility, has attracted attention from the very beginning of MRI via the presence of susceptibility artifacts (which cause signal dephasing near air/tissue interfaces and at the surfaces of metal implants). Outside of these bulk susceptibilities are the internal susceptibility differences between the tissues themselves. This has been used both to enhance contrast between tissues using susceptibility-weighted imaging (SWI) and to map the magnetic source characteristics using quantitative susceptibility mapping (QSM). Conventional SWI employs a fully flow-compensated GE acquisition to take advantage of both the intrinsic T2* decay from different biological sources and the phase from local changes in susceptibility to form an enhanced contrast image ^29^. It is used mainly to image the cerebral venous system in neuroimaging. QSM makes it possible to quantify susceptibility differences between tissues that can be caused by the level of blood oxygen saturation, iron deposition, calcification, de-/dysmyelination, and other internal structural variations that change the intrinsic susceptibility.

Imaging the PNS is technically feasible because there is little leg motion during scanning, and blood flow artifacts can be reduced by using flow compensation. However, pulsatility artifacts can still be a problem ^30^. For example, when imaging the cross section of the proximal sciatic nerve at the thigh level, one can use an anterior-posterior phase encoding to shift the arterial ghosting artifacts from left/right to up/down in an attempt to avoid corrupting the nerve. Compared with the brain, the peripheral nerve has much less complexity in its anatomy and function, which simplifies the optimization of image contrast. Particularly, the cellular and subcellular anatomy of the peripheral nerve makes DTI one of the best approaches to imaging the fascicles since the tube-like structure restricts molecular water motion to be mainly along the longitudinal nerve axons. However, there are also several limitations in imaging the peripheral nerve. First, anatomical imaging the fascicles and other structures requires high resolution, which takes longer and has a lower signal-to-noise ratio (SNR). Since the peripheral nerve runs parallel to the extremity, the axial orientation is typically used to investigate the cross-sectional morphometry. The nerve is only a few millimeters across and the fascicles are much smaller (on the order of 1 mm or less), except in some rare disorders like heredity neuropathy where the affected sciatic nerve fascicle at the distal-thigh level can be as large as 6 mm in diameter (unpublished observation). High-resolution imaging on the order of 0.25 mm or better will be needed to differentiate the signal from the neuron and from the intraneural lipid-equivalent tissues and minimize partial volume effects. Second, the peripheral nerve is always accompanied by fatty tissue which causes systemic error in quantitative measurements such as T1, PD, and T2 mapping; MT; fractional anisotropy (FA); and susceptibility. Therefore, peripheral nerve imaging typically employs fat saturation or water excitation pulses to minimize these problems. However, the use of fat saturation introduces its own problems and can affect quantification of the data and reduce the SNR. Third, there are limited sequences and software developed for imaging the peripheral nerve, making reliable interpretation of PNS difficult. Nevertheless, peripheral nerve MRI has grown recently, and a few potential biomarkers have been reported in various studies in patients with peripheral neuropathies ^17, 19, 21, 24, 25, 28, 31– 33^.

Diagnosing peripheral nerve diseases with MRI has been mainly case-based using conventional MRI sequences such as PDW, T1W, T2W, STIR, and gadolinium-enhanced T1W. For instance, in a study by Wasa *et al.*, conventional MRI was used to reveal mass lesions, peripheral enhanced lesions, edema, and intratumoral cystic lesion for differentiating malignant peripheral nerve sheath tumors from neurofibromas with a sensitivity of 61% and a specificity of 90% ^34^. Assessing infiltration could be very difficult, but it may be possible when there is a discrete fat appearance between the tumor and the peripheral nerve ^35^. T2W and diffusion MRI have depicted high signal intensity and plexus enlargement, suggesting inflammatory changes in patients with brachial plexopathies ^36^. For diseases in the phrenic nerve, MRI could be the preferred imaging technique since the nerve is difficult to access with other imaging or diagnostic methods ^36^. However, there is a lack of systematic investigation on specific diseases.

### Magnetic resonance neurography

Magnetic resonance neurography (MRN) plays a significant role in today’s clinical and scientific imaging of the peripheral nerve. The earliest work in using MRN are those done by Howe, Filler and their colleagues in the 1990s ^37, 38^. MRN depicts a nerve-only image with the basis of suppressing signal from other static tissues and blood vessels ^32, 37, 39^. The cross-sectional fascicular isolation of sciatic nerves was also described in early work by Filler *et al*. ^38^, who used the fact that the nerve has a longer T2 relaxation time compared with muscle. By combining heavy T2 weighting, diffusion weighting, and strong fat suppression as well as a spatial saturation band to eliminate in-flow effects inside major blood vessels, nerve-only images were generated with high nerve-to-background contrast.

Nerves in the lower and upper extremities as well as in the brachial and lumbosacral plexuses have been studied by using MRN, particularly in patients with either hereditary or acquired neuropathies. MRN demonstrated significant nerve enlargement in CMT1A compared with controls, but minimal nerve thickening in CMT2. There were T2 hyperintensities in both disease types ^20, 22, 24, 40, 41– 43^. Local or whole-body MRN also revealed significant nerve enlargement and T2-weighted signal increases in patients with CIDP and diabetic neuropathies ^20, 23, 25, 26, 44^. By measuring the cross-sectional area (CSA) and the signal intensity on T2W, Kronlage *et al*. reported diagnostic accuracies of area under the receiver operating characteristic curve (AUC) of 0.88 when using CSA in lumbosacral plexus and 0.88 when using T2W in sciatic nerve at the middle-thigh level ^23^. A study by Jende *et al*. quantified differences of microstructural nerve damage between type 1 and type 2 diabetes (T1D and T2D) using MRN ^26^. T2-weighted hypointense and hyperintense territories of the tibial and peroneal nerves were segmented manually and characterized to be predominantly in T1D and T2D, respectively. Correlations of MRN with other clinical measurements were weak to moderate but not strong. This study used adjacent muscle as a signal intensity reference to segment the two portions of nerve signals to be hyperintense or hypointense relative to muscle. This kind of quantification could be useful when conventional tissue property quantification is not available. Those hypointense voxels on the fat-saturated magnitude image suggest intraneural lipid aggregations or depositions, which do not directly affect nerve conduction since they are not related to demyelination or axonal degeneration ^26^.

Although MRN offers superb nerve-to-background contrast, leading to an increasing use of it in various peripheral disorders, there is still the need to calibrate the images across scanners in multi-center studies. On the other hand, it is difficult to use these signal intensity-based measurements to differentiate the two major fundamental pathologies: demyelination versus axonal loss. The combination of DWI and DTI could permit enough sensitivity to reveal pathological changes.

### Diffusion-weighted and diffusion tensor imaging

DWI and DTI have been two of the major techniques in neuroimaging used to explore the molecular water motion driven by thermal agitation and whose results are dependent on the tissue’s microstructure. In a typical DWI acquisition using an SE sequence, the pair of strong gradient lobes on either side of the 180° refocusing RF pulse make those diffusing protons not fully refocused. This leads to an exponential signal loss greater than what naturally occurs due to T2 decay for stationary protons. The apparent diffusion coefficient (ADC) for a particular direction, which describes the mobility of the molecules, can be measured by two signals acquired with and without using the diffusion gradient lobes ^45^. When the diffusion gradients are used in multiple directions (at least seven acquisitions, including six diffusion-weighted acquisitions with different gradient directions and one baseline image without diffusion gradients), the 3×3 symmetric matrix of diffusivity can be computed to extract eigenvalues along each coordinate axis. The FA represents the diffusion asymmetry within a voxel ^46, 47^. Axial diffusivity (AD) and radial diffusivity (RD) are defined as the major eigenvector direction and the mean of the other two minor eigenvalues, respectively. AD indicates water movement along the major direction of the tissue, whereas RD measures the water diffusion restriction perpendicular to the major direction. Furthermore, the fibers can be tracked in three dimensions (3D) by using the DTI eigenvectors to display water motion along the nerves ^48^. The FA values are high (close to unity) along the peripheral nerve because of the restricted water diffusion along the axonal direction as guided by the epineurium, perineurium, endoneurium, and myelin sheath in myelinated axons. Because of the predominantly unidirectional nature of the nerve fibers, fewer diffusional gradient directions are required in peripheral nerve DTI than in the brain for quantifying white matter fiber tracts ^49^. In the 1990s, peripheral nerve anisotropy was overserved in works on human sciatic nerve ^50^ and tibial nerve ^51^. More recently, Zhou *et al*. have reported that three major peripheral nerves in the forearm (ulnar, superficial radial, and median nerves) can be unequivocally visualized by high-resolution DTI ^52, 53^. To date, DTI has been involved in most of the studies in peripheral nerve MRI in patients with peripheral neuropathies. Chhabra *et al*. suggested that FA values are more useful than mean ADC values measured on cervical nerve roots in patients with CMT diseases compared with controls ^22^. Lichtenstein *et al*. also reported significantly decreased FA of sciatic nerves in patients with CIDP than controls ^25^. But there were no significant changes of FA at 6-month follow-up compared with baseline in the CIDP cohort, suggesting the slow progression of this type of disease ^25^. In a study by Vaeggemose *et al.*, decreased FA and increased ADC were statistically significant for both distal and proximal nerves in patients with CMT1A than controls ^24^. The use of ADC and FA can provide an understanding of the source of signal change in the presence of demyelination or axon loss. It has been reported that the non-myelinated nerves have anisotropy similar to that of myelinated nerves measured by diffusion MRI ^54^. Most likely, the strong anisotropy of nerve fibers is caused by the large axon density and inherent axonal membranes but not the presence of the myelin sheath, leading to a restriction of water motion mainly along the long axis of the nerve ^54^. Establishing the normal reference values of peripheral nerve anisotropy and diffusivity will be valuable in understanding the nerve development over time and in cohort-based multi-center studies. Kronlage *et al*. reported that FA values of all peripheral nerves in the extremities declined with increasing age and were inversely associated with body height, weight, and body mass index (BMI) ^55^. This suggests that age and BMI should be considered in studies using DTI in peripheral nerves. DTI has also been used to detect restriction-induced microscopic anisotropy in a single voxel to predict the average axon diameters (typically in less than 20 microns) using a mathematical model ^56^. This model could be very useful in predicting average axon diameter–associated nerve conduction velocities when nerves in deep tissues are not accessible by electrophysiological tools ^56^. However, further studies will be needed to prove the linear relationship between DTI-derived axon diameters and the nerve conduction velocities measured by NCSs.

### High-resolution fascicular magnetic resonance imaging of peripheral nerve

**Figure 2.**
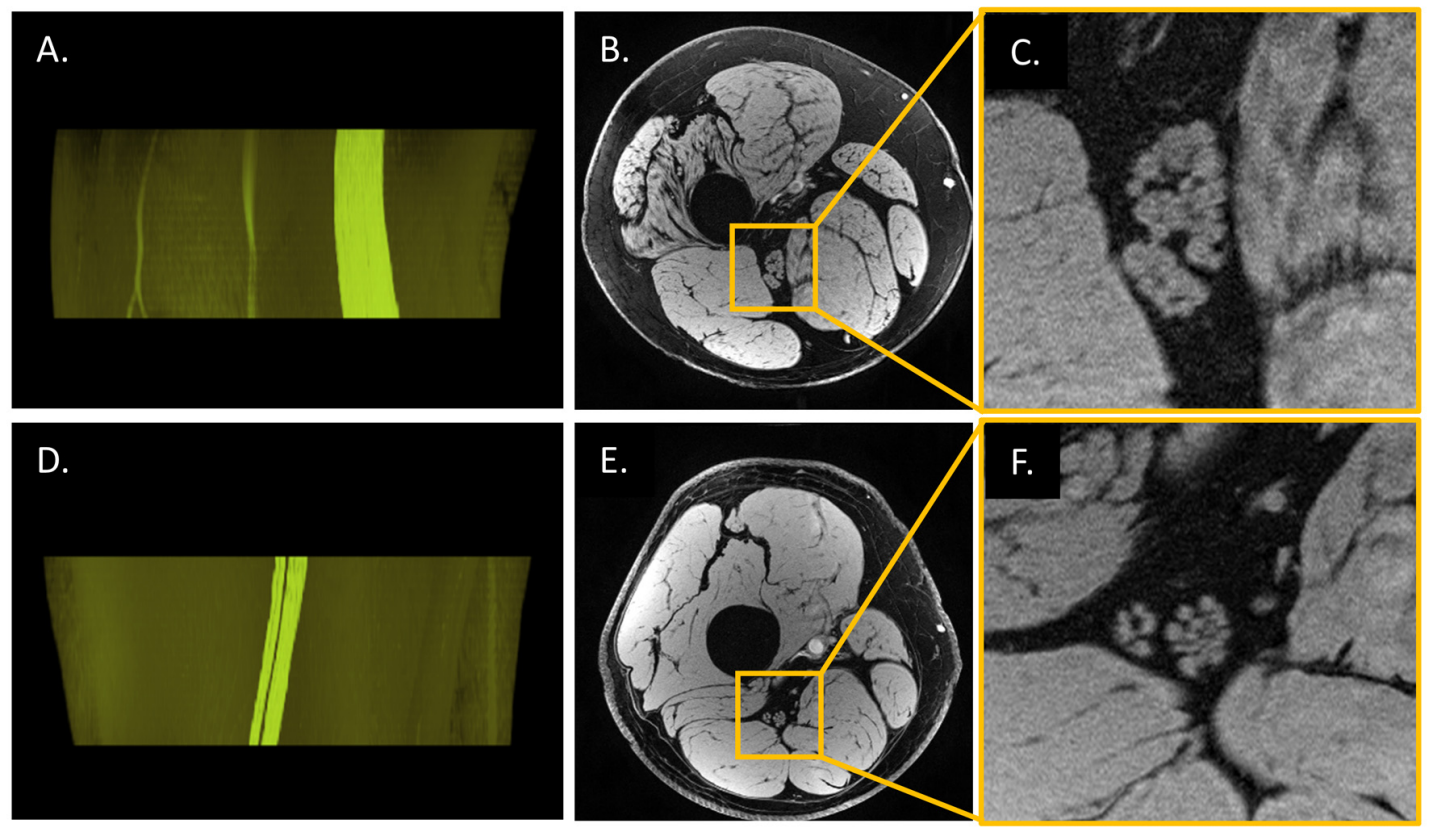
*In vivo* ultra-high-resolution magnetic resonance imaging of sciatic nerve in a patient with Charcot–Marie–Tooth disease (CMT) and healthy control. Images of a patient with CMT type 4J ( **A**– **C**, 35 years old, male) and those of a healthy control ( **D**– **F**, 35 years old, male) were acquired at distal 30% of femur length by using a three-dimensional (3D) high-resolution gradient-recalled echo scan with a voxel size of 0.15 × 0.2 × 3 mm ^3^; 3D fascicular nerve reconstructions ( **A** and **D**) were rendered (VolView 3.4, Clifton Park, NY, USA) from the overlay of the manually segmented tibial and peroneal portions of the nerve fascicles onto the original magnitude images ( **B** and **E**). The rightmost images ( **C** and **F**) were enlarged from **B** and **E**, respectively. CMT4J is a rare subtype of the inherited neuropathy caused by recessive genetic mutations with the loss of FIG4 protein which results in demyelination in peripheral nerves ^64^. Even though the significantly enlarged sciatic nerve cross-sectional area is a change in a number of peripheral neuropathies, it is not possible to differentiate demyelination versus axonal degeneration using the magnitude images (or other forms of conventional imaging such as proton density–weighted, T1-weighted, or T2-weighted imaging). However, susceptibility-based techniques such as T2* mapping, susceptibility-weighted imaging, and quantitative susceptibility mapping may be used to probe the integrity of the myelin. Imaging parameters were those listed in the fourth scan in Table 2.

**Figure 3.**
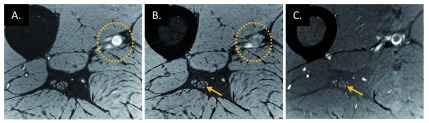
Differentiating intraneural blood vessels from nerve fascicles. High-resolution water-excited three-dimensional (3D) gradient-recalled echo scans of a healthy volunteer (38 years old, male) without ( **A**) and with ( **B**) spatial saturation bands placed on the proximal side of the imaging slab, which suppresses the signal of major arteries flowing into the imaging slab. However, spatial saturation pulses on the 3D acquisition slab work for fast flow (dotted circles on **A** and **B**) but not slow flow (arrows on **B** and **C**) that presents in the artery inside the epineurium. This interpretation of slow blood flow suppression is confirmed by using a thin slice acquisition ( **C**) which was from a 2D proton density–weighted scan with fat suppression using a turbo spin echo sequence.

High-resolution fascicular nerve imaging has been of great interest in the field for many years. The early work of Filler *et al*. two decades ago represented clear individual nerve fascicles on the distal sciatic nerve ^38^. Benefitting from the improved hardware and new imaging approaches, more studies have used high-resolution imaging in conjunction with better contrast methods to separate nerve fascicles from surrounding fatty tissues and the neural component from the perineurium ^33, 57– 61^. In a recent study by Felisaz *et al*. ^59^, a small field of view (6×6 cm ^2^) was used to acquire 100-micron in-plane resolution of the distal tibial nerve at the ankle level using a surface coil. The number of fascicles, nerve fascicular area, and epineurium area were computed to correlate with clinical features in patients with CIDP. Measuring MR signals predominantly from nerve fascicles with reduced partial volume effects potentially gives more accuracy in nerve morphometrics. Using 7T scanners can depict the fascicular structures better than 3T scanners because of the increased SNR ^33^. However, 7T is not broadly available at this moment. Furthermore, the RF field inhomogeneity corrections in 7T are more difficult than those in 3T. These corrections are essential for collecting quantitative data such as T1, PD, and T2 maps. A practical way to improve the image quality in ultra-high-resolution imaging is to use a better receiver coil with more coil elements (or receiver channels). That also gives the opportunity to increase the parallel imaging acceleration factor to reduce imaging time. Although the size of the coil elements decreases when there are many coils, new flex coils with 32- and 64-channel coils can be wrapped around limbs maintaining high SNR. Also, the use of 3D spoiled gradient-recalled echo (GRE) sequence leads to increased SNR relative to 2D imaging. By employing water excitation pulses or other fat suppression techniques, one may obtain high-resolution images depicting individual nerve fascicles with suppressed fat signal and providing a better 3D nerve fascicular reconstruction ( Figure 2). The same 3D GRE sequence can be run with several flip angles to correct the B _1_^+^ field and produce homogeneous T1, PD, and T2* maps for nerve, fat, and muscle ^62, 63^. Intraneural blood vessels should also be considered in case of fascicular quantifications. With spatial saturation bands, the slow flow in intraneural blood vessels might not be suppressed as expected in 3D GRE images ( Figure 3). These intraneural blood vessels may be distinguishable from the quantitative maps.

**Table 2.** A multi-contrast multi-parametric sciatic nerve imaging protocol at 3T.

Sequence	Ori.	TR, ms	TEs, ms	Res., mm ^2^	FA, deg	NS	Nex	TH, mm	BW, Hz/px	TA, m:s	Comments
2D GRE	COR	7.5	3.3	1.8 × 3.6	50	13	3	6	320	0:30	First two scans are for femur length measurement using spine coil
2D GRE	COR	7.5	3.3	1.8 × 3.6	50	13	3	6	320	0:30	
2D GRE	T/C/S	7.7	3.7	1.1 × 1.5	20	3/5/9	1	6	300	0:30	Knee coil localizer to locate sciatic nerve and central slice
3D GRE	TRA	26	5.1	0.15 × 0.2	12	40	1	3	110	7:30	Ultra-high resolution with water excitation for nerve fascicular segmentation
2D TSE	TRA	5000	15, 77	0.6 × 0.6	180	20	4	3	440	5:27	Dual-echo 2D scan for T2 mapping without slice gap
3D GRE	TRA	20	7.6, 8.85	0.3 × 0.3	20	40	1	3	150	6:12	Interleaved dual-echo scans for water/fat imaging
3D GRE	TRA	30	5.1, 20.1	0.3 × 0.3	5	40	1	3	210	5:57	These two GRE scans are for T1, proton density mapping with the extraction of B _1_^+^ and B _1_^−^ field maps
3D GRE	TRA	30	5.1, 20.1	0.3 × 0.3	30	40	1	3	210	5:57	
3D GRE	TRA	35	10.1	0.8 × 0.8	15	40	1	3	110	2:46	These two scans with and without magnetization transfer pulse are for the magnetization transfer ratio calculation
3D GRE	TRA	35	10.1	0.8 × 0.8	15	40	1	3	110	2:46	
ssEPI	TRA	5400	93	1.2 × 1.2	180	20	3	3	1400	6:09	20 direction diffusion tensor imaging scan with b = 0/1000 s/mm ^2^

All transverse scans have the same field of view (154 × 154 mm ^2^). 2D, two-dimensional; 3D, three-dimensional; BW, bandwidth; COR, coronal; FA, flip angle; GRE, gradient-recalled echo; NEX, number of average; NS, number of slices; Ori., orientation; Res., acquisition resolution; SAG, sagittal; ssEPI, single short echo planner imaging; T/C/S, three-plane; TA, time of acquisition; TE, echo time; TH, slice thickness; TR, repetition time; TRA, transverse; TSE, turbo spin echo.

### Magnetization transfer

Generally speaking, biological tissues consist of two populations of water protons: bound water protons in macromolecules and free water protons. The former has much shorter T2 than the latter because of the restricted motion and has a broad range of resonance frequency offsets relative to the Larmor frequency of free water protons. When an off-resonance RF preparation pulse is used, the bound water can be selectively saturated. Thereby, this pre-saturation of bound water suppresses the free water protons’ magnetization vector because of the water exchange between them. This is referred to as magnetization transfer contrast (MTC) ^65, 66^. The signal decrease ratio is thereby referred to as the magnetization transfer ratio (MTR), which is calculated from the signal differences and normalized by the unsaturated signal (M _0_) as MTR = (M _0_−M _T_)/M _0_, where M _T_ is the off-resonance signal. MTR has been widely used in characterizing subtypes of white matter lesions in patients with multiple sclerosis ^67^ because the MTR changes of normal-appearing white matter (NAWM) are predominantly from the presence of myelin in the shape of macromolecules. In the case of demyelination, the MTC signal increases in lesion territory compared with the myelinated NAWM, leading to a decreased MTR value. MT has also been used in musculoskeletal MRI and in imaging peripheral nerves in patients with peripheral neuropathies because of its sensitivity to the myelin density changes caused by either demyelination or axonal loss ^19, 21, 67– 70^. Dortch *et al*. reported promising results that MTR values on proximal sciatic nerve were significantly decreased in patients with CMT relative to those from controls, suggesting a viable biomarker of demyelination or axonal degeneration (or both) in these disorders ^19^. The study employed a pair of 3D multi-shot echo-planar acquisitions at the middle-thigh level. Apparent MTR values were computed with the correction of B _1_^+^ field variations. These MTR measurements were also significantly correlated with disability scores for all patients. However, there was no statistically significant difference of MTR observed between CMT1A and CMT2. Nevertheless, MTR could be a viable tool to reveal changes resulting from the combination of demyelination and axonal loss. Longitudinal studies are expected to probe disease progression or treatment response using this technique. One of the potential pitfalls in multi-center studies could be the calibration of MTR values derived from different types of scanners. This semi-quantitative parameter varies largely from different MT pulse parameters as well as the B _1_^+^ field variation correction efficiency ^68^.

### Thermal relaxation quantifications

As discussed in previous sections, the differences of T1 and T2 relaxation rates as well as the PD and T2* from different tissues determine the contrasts in conventional images but their quantitative values can be the key to find specific biomarkers in various neurodegenerative diseases. These quantitative measurements are very much underutilized in imaging the peripheral nerves. Several studies used quantitative T2 and PD of the peripheral nerve derived from two or more echoes of the fast SE sequence ^24, 21, 59, 61, 71, 72^. With these multiple acquisitions using different TEs, one can quantify T2 and T2* relaxation rates as well as PD by fitting the signal dependence for that sequence. To quantify T1, one can use an SE sequence with multiple inversion recovery (IR) times to do the curve fitting of the longitudinal magnetization. However, this multi-IR method is time-consuming given the need of a long TR for reliably measuring long T1 and acquiring multiple slices. In their study involving patients with CMT1A and healthy controls, Vaeggemose *et al*. used 10 echoes to quantify T2 and PD of sciatic and tibial nerves in the lower limbs ^24^. PD values of sciatic nerve were significantly higher in patients with CMT1A than controls, and there was no statistically significant difference between the two groups for the T2 measurements in both the sciatic and tibial nerves. T2 values reported in this study on distal nerves were different from but close to those in the recent study by Felisaz *et al*. ^59^. Minimizing the disturbances caused by intraneural fatty tissues using high-resolution acquisitions would reduce quantitative variations significantly. Nevertheless, these studies using quantitative tissue properties demonstrated promise for providing new information in imaging the peripheral nerves.

Alternatively, 3D GE-based variable flip angle methods have been a major branch of T1 mapping but require knowledge of the B _1_ field variations, especially at 3T and higher magnetic field strengths. Various B _1_ mapping methods ^62, 73, 74^ can be used in imaging the sciatic nerve in the mid-thigh level. With the knowledge of transmit and receiver B _1_ field variations, T1 and PD can be computed in a pixel-by-pixel manner. However, to the best of our knowledge, *in vivo* T1 mapping and accurate true PD mapping as well as QSM in patients with peripheral neuropathies have not been investigated at this point. These uncultivated quantifications may reveal more viable biomarkers in assessing disease progression in terms of monitoring water changes caused by either metabolic or pathologic changes. Susceptibility mapping may help in differentiating the two fundamental pathologies: demyelination from axonal degeneration.

### Chemical shift imaging

**Figure 4.**
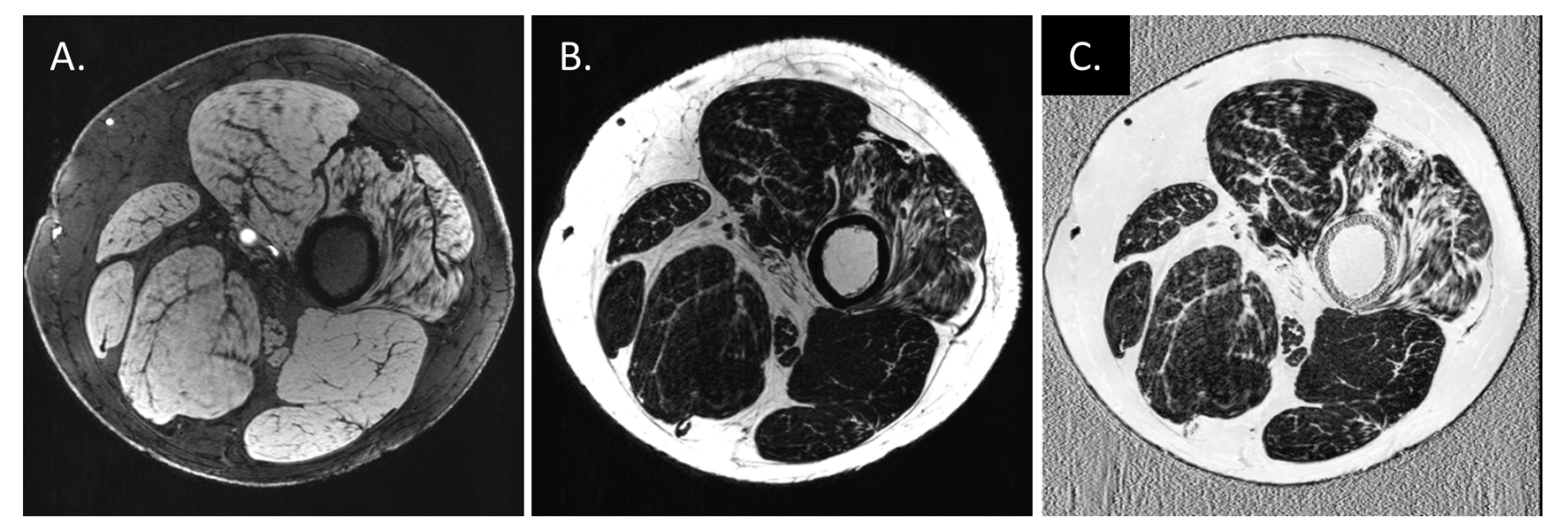
Interleaved two-point Dixon water/fat separation. Images were from the same patient with Charcot–Marie–Tooth disease type 4J (35 years old, male) using an adapted gradient-recalled echo sequence ^86^. Two echoes with echo times of 7.6 ms (in-phase) and 8.5 ms (out-of-phase) were acquired in an interleaved manner so that both images are naturally co-registered to each other. Phase ambiguities were resolved by using a projected power method in the two-point Dixon water/fat separation to get water (W, **A**) and fat (F, **B**) images ^81^. The fat fraction (FF) ( **C**) was computed to be FF = F / (W + F) after shifting the fat image to its real position by n pixels in the readout direction, where n = imaging frequency × 3.5 / bandwidth. Muscle atrophy with increased FF can be observed on the right-most muscle in this case.

The presence of fatty tissue surrounding most of the peripheral nerves as well as the intraneural lipid-equivalent connective tissues makes quantifying or eliminating fat from the image critical since intramuscular or intraneural fat accumulation is one of the most common pathological processes in neuromuscular disorders and peripheral neuropathies. Several fat suppression methods that take advantage of the frequency difference between water and fat (roughly 3.5 parts per million) have been used in peripheral nerve imaging. Moreover, fatty tissue has longer T2 and shorter T1 than either muscle or nerve tissue. In terms of suppressing the fatty tissue, one can use a preparation RF pulse tuned to the fat resonance frequency to saturate fatty tissues or tuned to the water signal to selectively excite water protons ^75^. One can also use a STIR preparation pulse to null the signal from fatty tissues ^76^. With the use of adiabatic RF pulses, STIR is much less sensitive to the B _0_ and B _1_ field inhomogeneities than those approaches using selective frequency pulses. Alternatively, with a slight shifting of the TE to be water and fat in-phase or out-of-phase, two or three of these acquisitions can be used to acquire a water-only image along with a fat-only image, known as Dixon methods ^77^, and many variants of this approach exist ^78– 80^. The various Dixon methods require knowledge of the B _0_ variations to properly separate water from fat signals ^81^. The water faction and fat fraction (FF) can be derived from these water-only and fat-only images. Particularly, FF = F/(W+F) is used in musculoskeletal MRI, as shown in Figure 4. Increased muscular FF reflecting intramuscular fat accumulation which is a common downstream effect of denervation in peripheral neuropathies and has been shown to be a valuable biomarker for the progression in a number of neuromuscular disorders ^21, 25– 26, 82, 83^.

Morrow *et al*. investigated a few quantitative MRI measurements on cross-sectional images of muscles, including FF, T2 maps, and MTR, to determine the responsiveness of these MRI measures and their correlations to clinical measures in a prospective longitudinal cohort with genetically confirmed CMT1A and inclusion body myositis (IBM) ^21^. Each subject had two visits: one at baseline and one at a 1-year follow-up to observe progression of disease. The study indicated that the cross-sectional whole muscle FF increased significantly during the 1-year follow-up at the calf level but not at the thigh level in patients with CMT1A and at both levels in patients with IBM. Their results correlated to clinical measures. T2 (increases) and MTR (decreases) changed consistently with FF increases in muscle. Compared with the control group, patients with CMT1A had increased FF and T2 and decreased MTR at the thigh and calf level, but only the first two were statistically significant. Considering these findings, the authors suggested that FF could be a responsive *in vivo* measure to monitor intramuscular fat accumulation in neuromuscular disorders. For inherited and acquired peripheral nerve disease, intramuscular fat accumulation is the final pathological change that takes place secondary to axonal loss; thus, the FF, T2, and MTR in muscle provide an indirect measure of axonal loss. Therefore, finding a better biomarker directly assessing peripheral nerve pathologies is still a pressing issue.

### Post-processing approach

**Figure 5.**
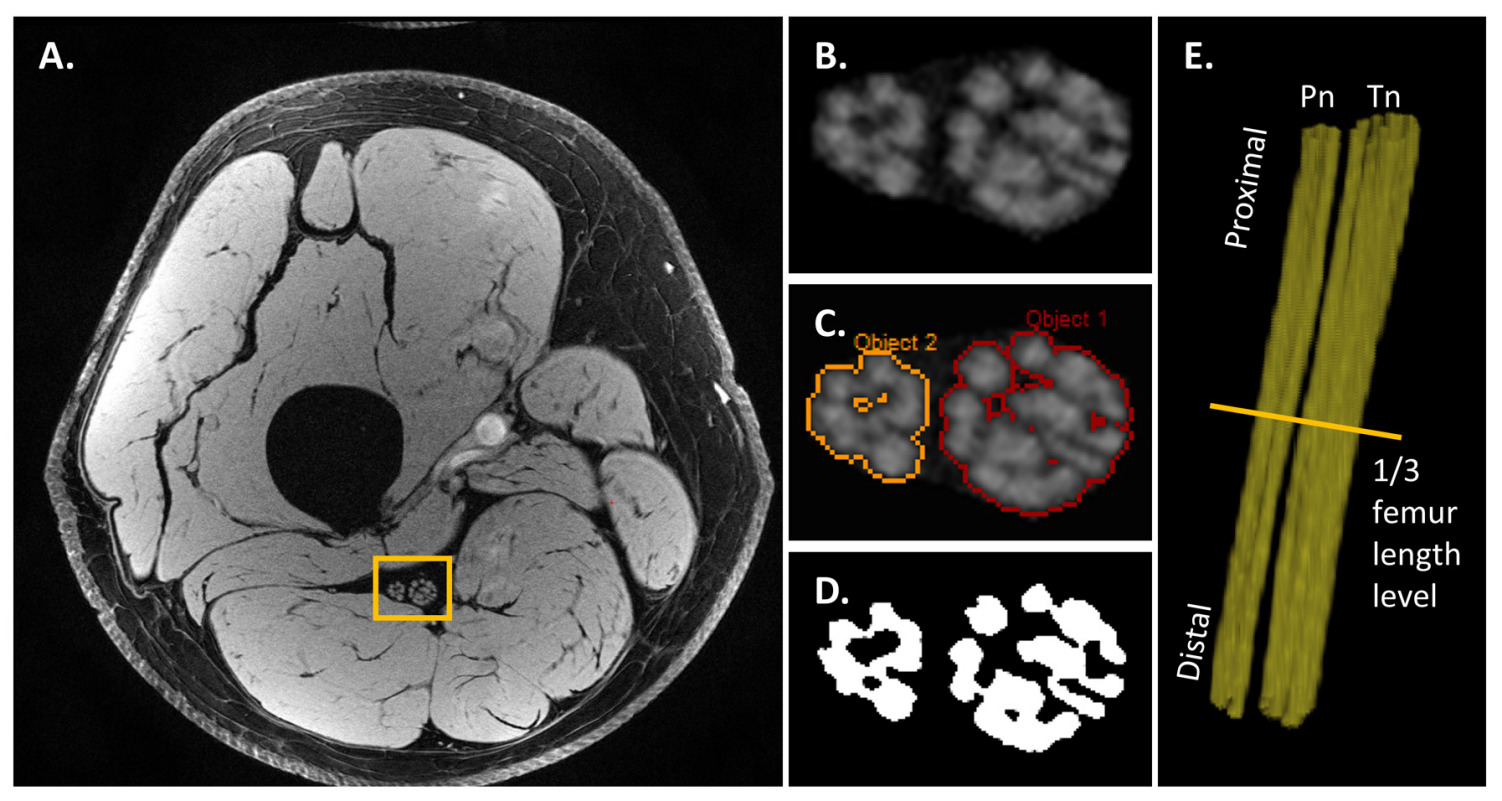
Semi-automated sciatic nerve fascicle segmentation and three-dimensional (3D) reconstruction. This representative data was acquired using a high-resolution 3D gradient-recalled echo with water excitation on a healthy volunteer (35 years old, male). The sciatic nerve areas ( **B**) were manually drawn by an experienced neuroradiologist on the original magnitude image ( **A**). Then the nerve fascicles ( **C**) were extracted by using a histogram-based region growing approach (SPIN software, MR Innovation, Bingham Farms, MI, USA). The binary masks ( **D**) of nerve fascicles were generated with pixel erosion. The nerve fascicular 3D reconstruction ( **E**) (Pn, peroneal nerve; Tn, tibial nerve) was then generated by using 3D rendering (VolView 3.4, Clifton Park, NY, USA).

Segmenting peripheral nerves as well as nerve fascicles provides one step toward quantifying the nerve morphometries derived from MRI. Such measures might include nerve volume, fascicular volume, fascicle-to-nerve volume ratio, and nerve CSA. Nerve fascicular binary masks derived from ultra-high-resolution anatomical acquisitions could increase the accuracy of other quantifications, such as T1, T2, and PD mapping and MTR, when the data are acquired in the same MRI session and are properly co-registered. One potential pitfall is the imperfection of image co-registration, which increases the variation of quantitative data from multiple scans. The reason is that, unlike the brain restricted by the skull, peripheral nerve and surrounding soft tissues could be distorted during the MRI session. These segmentations are usually performed manually or semi-automatically by experienced neuroradiologists ^26, 57^. With fat suppression and high-resolution data acquisition, T1W or T2W images depict individual nerve fascicles well. Therefore, one can manually tailor the nerve areas from the original magnitude images. When histogram-based region grow algorithms are used with proper pixel erosion, nerve fascicular binary masks can be extracted easily ( Figure 5). These semi-automated methods have tolerance to signal variations caused by B _0_ and B _1_ field inhomogeneities. Jende *et al*. also used the adjacent muscle area as a reference threshold to segment hypointense and hyperintense regions inside the epineurium on T2W fat-suppressed images ^26^. This reference value eliminated the need for bias field correction, making the results more reliable. Recently, deep learning–based fully automatic approaches demonstrated promising results on segmenting peripheral nerve, nerve fascicles, and axons ^85, 86^. Moiseev *et al*. used a convolutional neural network (CNN)-based approach to segment nerve axons and myelin thickness on semithin section images of mouse sciatic nerve ^86^. In contrast, the use of MRI images to segment human peripheral nerve and nerve fascicles obviously needs more *in vivo* data acquired from a standardized protocol to train the CNN-based approaches and more research effort as well.

## Recommendation for peripheral nerve magnetic resonance imaging

### Practical three-dimensional imaging of the sciatic nerve

High-resolution tissue quantification at the fascicular level has great potential to probe pathophysiological biomarkers in peripheral nerves. Establishing the quantitative properties for T1, PD, T2, T2*, MTR, diffusional parameters, and susceptibility will likely help elucidate normal development and pathophysiological changes in peripheral neuropathies. Revealing proximal pathology is key to study disease progression and responsiveness to therapeutic interventions because distal leg nerves are often degenerated in patients with chronic peripheral neuropathies, thereby resulting in a “floor effect”. For instance, imaging and quantifying the proximal sciatic nerve at the thigh level could be an excellent approach in patients with CMT.

The protocol recommended in Table 2 can be acquired in roughly 45 minutes by using a knee coil at 3T. Using this imaging protocol, one can exactly localize the cross-sectional level of the nerve for each axial slice with respect to the femur length. The ultra-high-resolution GRE scan provides 3D images to visualize and segment sciatic nerve fascicles. The two-point Dixon water/fat separation gives the ground truth of water and fat tissues with the in- and out-of-phase images acquired in a naturally co-registered manner ^84^. With this water/fat segmentation data, the variable flip angle T1 and PD mappings can be corrected for RF field variation ^62, 63^. These B _1_^+^ and B _1_^−^ field maps also serve as the calibration of MTR. The multiple echoes from the GRE scans can be used to compute the T2* decay and susceptibility of neuronal changes with respect to surrounding tissues.

## Conclusions

Various MRI methods have been investigated in peripheral neuropathies ( Table 1). Even though muscle atrophy is commonly seen in peripheral neuropathy, the direct assessment of peripheral nerves is still required to access nerve pathologies. Nerve enlargement is a frequent change in a number of peripheral neuropathies. Increases of T2W and ADC and decreases of MTR and FA of peripheral nerves have been reported in patients with CMT, CIDP, and DPN. High-resolution fascicular quantifications are promising but need more systematic studies to translate into clinical tools. Methods for probing the proximal nerve are important to study disease progression and treatment responses. There is also a need for reliable imaging biomarkers to distinguish the two fundamental types of nerve pathology: de-/dysmyelination versus axonal degeneration. QSM may be able to reveal susceptibility changes between myelinated and un-myelinated tissue ^87^. The QSM of the peripheral nerves needs further development to address a couple of issues introduced by the complicated phase behavior in the nerve territory when using a long TE and the interference from the intraneural lipid-equivalent connective tissues.

## Ethics

Written informed consent forms were obtained from the patients in Figure 2, Figure 3, Figure 4 and Figure 5 for the use and publication of these images. The authors also obtained approval by the local institutional review board. **
